# Identification of SARS-CoV-2 Main Protease Cleavage Sites in Bovine β-Casein

**DOI:** 10.3390/ijms26125829

**Published:** 2025-06-18

**Authors:** János András Mótyán, Tibor Nagy, Ágota Nagyné Veres, Mária Golda, Mohamed Mahdi, József Tőzsér

**Affiliations:** 1 Department of Biochemistry and Molecular Biology, Faculty of Medicine, University of Debrecen, 4032 Debrecen, Hungary; averes@med.unideb.hu (Á.N.V.); golda.maria@med.unideb.hu (M.G.); mohamed@med.unideb.hu (M.M.); 2 Department of Applied Chemistry, Faculty of Sciences and Technology, University of Debrecen, 4032 Debrecen, Hungary; nagy.tibor@science.unideb.hu

**Keywords:** SARS-CoV-2, main protease, coronavirus, casein, protease, cleavage site, substrate specificity

## Abstract

The severe acute respiratory syndrome coronavirus 2 (SARS-CoV-2) is the causative agent of the coronavirus disease of 2019 (COVID-19) and has persistently caused infections since its emergence in late 2019. The main protease (Mpro) of SARS-CoV-2 plays a crucial role in its life-cycle; thus, it is an important target for drug development. One of the first virus-specific drugs that has been approved for the treatment of COVID-19 patients is Paxlovid, which contains nirmatrelvir, a covalent inhibitor of Mpro. Screening of inhibitor candidates and specificity studies also rely on efficient substrates and activity assays. Casein is one of the most commonly applied universal substrates that can be used to study a wide range of proteases, including SARS-CoV-2 Mpro. Casein is a known substrate for Mpro in vitro, but the specific casein isoform cleaved by Mpro remained unidentified, and the cleavage sites have yet to be determined. This work studied cleavage of α-, β- and κ-isoforms of bovine casein by SARS-CoV-2 Mpro, using in vitro and in silico approaches. The candidate cleavage sites were predicted in silico based on the protein sequences, and the cleavage positions were identified based on mass spectrometric analysis of cleavage fragments. Based on our results, only β-casein contains cleavage sites for Mpro and thus can be used as its substrate in vitro. The newly identified cleavage site sequences further widen the knowledge about the specificity of SARS-CoV-2 Mpro.

## 1. Introduction

The severe acute respiratory syndrome coronavirus 2 (SARS-CoV-2) is the causative agent of the coronavirus disease of 2019 (COVID-19). Since the outbreak of the pandemic in December 2019, the World Health Organization (WHO) has reported >777 million confirmed infections and >7.0 million COVID-19-related deaths (till June 2025). Although COVID-19 no longer constitutes a public health emergency of international concern (PHEIC), research on SARS-CoV-2 remains of special importance, particularly in areas such as clinical treatments, vaccine development and inhibitor design.

The genome of SARS-CoV-2 encodes two cysteine proteases, a papain-like (PLpro) and a main protease (Mpro). The main protease is also referred to as 3-chymotrypsin-like protease (3CLpro). Both PLpro and Mpro contribute to the limited proteolysis of the viral polyprotein, releasing functional proteins [1]. Due to its essential role in the viral life-cycle, SARS-CoV-2 Mpro has become one of the most important targets of antiviral drug development [2], and it has also been extensively studied in response to the growing resistance against the protease inhibitors [3]. Since the beginning of the COVID-19 pandemic, numerous mutations of SARS-CoV-2 have been identified [4,5]. Periodic viral genomic sequencing helps to detect new genetic variants circulating in communities [4]. An updated version of the SARS-CoV-2 phylogenetic tree is shared on the GISAID platform (Global Initiative on Sharing Avian Influenza Data). A variant is recognized as a Variant of Concern (VOC) or Variant of Interest (VOI) by the World Health Organization (WHO) [4].

Investigation of SARS-CoV-2 Mpro’s enzymatic characteristics and efficacies of its candidate inhibitors relies on the activity assays for which various peptide and protein substrates have been designed so far. Some of the substrates are specific and represent a natural cleavage site sequence of SARS-CoV-2 Mpro, for example, peptide substrates conjugated with fluorogenic group [6,7,8,9,10] or fluorescent protein-containing recombinant protein substrates (His_6_-MBP-mEYFP) [11,12]. Non-specific substrates are also available, such as casein, which is one of the most commonly applied universal substrates for protease activity assays [13,14]. While specific substrates enable targeted investigation of the protease activity, general substrates are considered as cheap, easy-to-use and efficient tools to investigate various proteolytic enzymes by using simple experimental setups.

Multiple non-viral proteins may contain sequences that closely resemble viral protease cleavage sites (including those of SARS-CoV-2 proteases) [11,15,16,17,18]; therefore, they can serve as candidate substrates for the viral proteases. The general protease substrate casein and asocasein are examples of such non-viral proteins that have already been used as substrates of the Mpro of SARS-CoV [19] and SARS-CoV-2 [20,21,22], as well as a fluorescein isothiocyanate (FITC)-conjugated form of casein [23]. Although bovine casein has already been reported to be processed by SARS-CoV-2 Mpro, the casein isoform(s) (α, β and/or κ) that serve(s) as a substrate for the protease and, additionally, the specific cleavage sites of Mpro have yet to be identified.

Interestingly, some studies using a casein-based assay described that SARS-CoV-2 Mpro results in the liberation of free tyrosine which can be measured spectrophotometrically [20,21,22]. Even though the liberation of free tyrosine aligns with the general description of the casein-based assay [13], it contrasts with the well-established specificity of SARS-CoV and SARS-CoV-2 Mpro. The Mpro is highly specific for Gln in P1 position [24,25]; however, His residue may also occupy this position in the minority of the known cleavage sites, both in case of SARS-CoV [26,27,28] and SARS-CoV-2 Mpro [29,30]. SARS-CoV-2 Mpro cleavage sites containing Tyr residue in the P1 position have not been described so far.

In this work, we aimed to investigate different isoforms of bovine casein as candidate substrates for SARS-CoV-2 Mpro, including identification of cleaved isoforms and the cleavage positions, by using computational and experimental approaches.

## 2. Results and Discussion

### 2.1. In Silico Prediction of SARS-CoV-2 Mpro Cleavage Sites in Casein

It has been previously reported that casein serves as a substrate of SARS-CoV-2 Mpro [20,21,22]; however, the specific casein isoforms that can be processed by the enzyme have not been identified, nor have the cleavage positions been determined. Therefore, we decided to analyze the sequences of α-S1-, α-S2-, β- and κ-isoforms of bovine casein in silico and predict cleavage sites of SARS-CoV-2 Mpro using NetCorona-1.0 and the 3CLP online tools. Both methods have already been successfully applied for the identification of Mpro cleavage sites [11,12,26,31,32]. It is important to note that these algorithms can only identify sequences containing Gln at the P1 position, although in rare cases, a His residue may also occupy the P1 position in SARS-CoV-2 Mpro cleavage [29,30].

**Figure 1:**
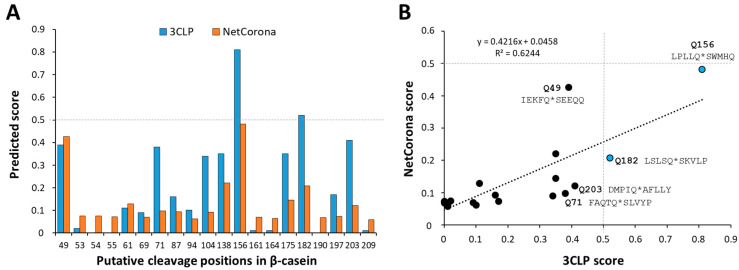
Prediction of SARS-CoV-2 Mpro cleavage sites in β-casein. The cleavage site prediction was performed using NetCorona-1.0 and 3CLP web servers. (**A**) The predicted scores are plotted for the possible cleavage positions of β-casein (UniProt ID: P02666). (**B**) The comparison of the scores predicted by the NetCorona-1.0 and 3CLP online tools was performed by linear regression analysis. Black and blue dots show data points. The blue dots show those 3CLP scores which were higher than the threshold, indicating putative cleavage at the given sites. Some of the putative cleavage site sequences are represented, asterisks indicate cleavage positions.

The predictions implied the presence of Mpro cleavage sites exclusively in β-casein (Figure 1A), while κ-, α-S1- and α-S2-isoforms were predicted to be absent from such sites (Table S1). The results of the two different predictions were in good agreement, but the predicted scores did not show a strong correlation (Figure 1B). A relatively higher number of cleavage sites was identified by 3CLP than by the NetCorona web server, similar to the former analysis of cleavage sites of human cellular proteins [12]. The cleavage probabilities calculated by the two methods showed considerable difference in the case of some positions (e.g., Q71, Q104, Q138, Q175 and Q203), and the scores obtained by 3CLP were higher compared to NetCorona (Figure 1A). Only the 3CLP algorithm identified the Q156 and Q182 as putative P1 residues with >0.5 prediction score in β-casein; the cleavage probability estimated by NetCorona 1.0 was lower, and the calculated scores were lower than 0.5. The probabilities of all putative β-casein cleavage sites were lower than the threshold when NetCorona 1.0 was used for prediction, which implied that β-casein might contain only few high-affinity cleavage sites based on this sequence analysis.

### 2.2. In Vitro Cleavage of Casein Isoforms with SARS-CoV-2 Mpro

In order to confirm the presence or absence of predicted Mpro cleavage sites in different casein isoforms, in vitro cleavage reactions were performed. We used different casein products, such as purified α- (containing α-S1 and α-S2 isoforms), β- and κ-caseins, and a casein fraction that was isolated from bovine milk and contained all of these isoforms.

**Figure 2:**
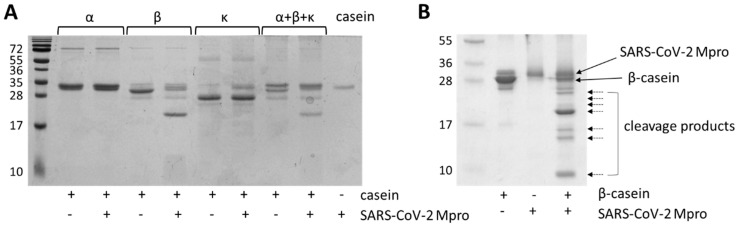
Cleavage of casein isoforms with SARS-CoV-2 Mpro. (**A**) A representative gel image shows processing of α-, β- and κ-casein isoforms with SARS-CoV-2 Mpro. (**B**) Cleavage of β-casein with SARS-CoV-2 Mpro. Arrows show SARS-CoV-2 Mpro and β-casein, while the casein cleavage products are shown with dashed lines.

The cleavage reactions revealed that β-casein is efficiently processed by SARS-CoV-2 Mpro, but cleavage was not observed for α- and κ-caseins (Figure 2A). The results of experimental cleavage reactions were in good agreement with those of the in silico predictions, which implied the presence of Mpro cleavage sites only in β-casein (Table S1).

β-casein was efficiently hydrolyzed by Mpro even in the presence of α- and κ-casein; therefore, a mixture of casein isoforms (with the αS1 and β isoforms in the highest amount) can also be used effectively for activity measurements.

### 2.3. Mass Spectrometry-Based Analysis of SARS-CoV-2 Mpro Cleavage Sites

The in vitro cleavage reactions revealed that only β-casein is processed by SARS-CoV-2 Mpro (Figure 2A). In order to identify the cleavage sites, reaction mixtures containing the proteolytic fragments (Figure 2B) were subjected for MALDI-TOF mass spectrometry (MS) analysis. We have already used this experimental approach successfully to determine the molecular masses of proteolytic fragments and identify proteolytic cleavage sites of SARS-CoV-2 Mpro [11,12] as well as the aspartic protease of the Ty1 retrotransposon of budding yeast *Saccharomyces cerevisiae* [33].

The cleavage positions were identified based on the comparison of the measured (determined by MS) and calculated (computed based on protein sequence) molecular weights of the proteolytic fragments. Molecular weights of β-casein and its cleavage fragments were calculated by considering the fact that the N-terminal signal sequence (1–15) is absent from the mature protein and β-casein undergoes phosphorylation at multiple serine residues (S30, S32, S33, S34 and S50). In addition, it is known that multiple sequence variants of β-casein exist (Table S2), with their occurrence showing characteristic differences across various breeds and countries [34]. The variant composition and the degree of phosphorylation of the applied β-casein was unknown; therefore, we used the canonical sequence of β-casein which was supposed to be phosphorylated at all five possible positions. A limitation of our predictions was that the applied in silico methods can only predict cleavage sites containing Gln in the P1 position, and they are not capable of identifying sequences with a P1-His residue.

The measured and calculated molecular weights are shown in Table 1. The molecular mass determined for β-casein experimentally was in agreement with its theoretical molecular weight, and it was comparable to that obtained previously for bovine milk casein [35,36]. The results implied that β-casein is cleaved by SARS-CoV-2 Mpro at the 71st, 87th, 94th, 156th, 182nd and 190th positions, next to P1-Gln residues in each case. Based on the results of MALDI-TOF MS analyses, the most preferred cleavages occur at the 71st, 94th, 156th and 190th positions of bovine β-casein. The cleavages at the other sites (at the 87th and 182nd positions) were more readily detectable if longer incubation time was applied (Figure 2B). Cleavage products indicating cleavage events next to tyrosine residues were not identified.

The 156th and 182nd positions were identified with the highest calculated scores by 3CLP as the most probable candidate sites. The 3CLP score of the 71st position was slightly lower than the 0.5 default threshold, but the 87th, 94th and 190th positions were predicted to have only a very low probability (Figure 1A). The sequence-based predictions implied the existence of other cleavage sites, such as the 49th and 203rd positions, that were also predicted to have relatively higher probability (Figure 1). As recommended, the structural characteristics were also considered while estimating cleavage probabilities [11,12,26,31]. Most of the predicted P1 residues were found to be located in extended regions of β-casein, but those which are buried or located within helical regions (e.g., 49th, 203rd) were considered to be less accessible to proteolysis (predicted P1 residues of β-casein are shown in Figure S1). In addition, post-translational modifications, such as phosphorylation, may also interfere with the proteolytic processing [37]. Cleavage at the 49th position was not observed, possibly due to the phosphorylation of the P1’-Ser residue of this cleavage site (S50). A possible limitation of this study is that the effects of cleavage site phosphorylation on the proteolysis were not studied in detail. However, all commercially available isolates are assumed to contain casein its phosphorylated form, preventing cleavage by Mpro at this site.

## 3. Materials and Methods

### 3.1. Proteins

The applied enzyme and the protein substrates used in this study are listed in Table 2. SARS-CoV-2 Mpro was expressed in its His_6_-tagged form. After affinity purification, the fusion tag was removed using factor Xa (FXa), followed by purification with ion-exchange chromatography [38].

### 3.2. In Silico Cleavage Site Prediction

SARS-CoV-2 Mpro cleavage sites were predicted based on protein sequences by using NetCorona-1.0 [24] and 3CLP (an online tool for predicting coronavirus 3CL protease cleavage sites) online web servers [25]. The 3CLP web server was applied on January 10, 2025 (http://www.computationalbiology.cn/3CLPHost/home.html (accessed on 10 July 2023)). The NetCorona-1.0 web server was available at the time of the analyses (July, 2023) (https://services.healthtech.dtu.dk/services/NetCorona-1.0/ (accessed on 10 July 2023)), but it was discontinued on/by January 2025, and only the downloadable version was available. Protein information was obtained from the UniProt database [39]. The default threshold of both NetCorona-1.0 and 3CLP was 0.5, and sites having <0.5 score were not considered to be cleaved. The higher the prediction score, the higher cleavage probability.

### 3.3. Cleavage Reactions Using Casein as Substrate

The casein isoforms that were used as substrates are shown in Table 2. The SARS-CoV-2 Mpro cleavage reactions were performed in a reaction buffer (50 mM NaCl, 20 mM TRIS-HCl, pH 7.5), in 48 µL final volume. The reaction mixtures were incubated at 37 °C for 60 min while shaking at 250 rpm, followed by separation of substrates and cleavage products by sodium dodecyl sulfate polyacrylamide gel electrophoresis (SDS-PAGE).

After cleavage reactions, the samples were prepared for electrophoresis under denaturing conditions: the samples were supplemented with 6× loading buffer (300 mM TRIS, pH 6.8, 20% glycerol, 0.05% bromophenol blue, 12% SDS, 100 mM β-mercaptoethanol) and then incubated at 95 °C for 10 min. The proteins were separated by SDS-PAGE (4% stacking and 14% resolving polyacrylamide gel). The gels were stained with Coomassie Brilliant Blue dye, and the gel imaging was performed using the Azure 600 imaging system (Azure Biosystems, Dublin, CA, USA).

Densitometry was performed using GelAnalyzer 2010a (www.gelanalyzer.com (accessed on 10 July 2023)) which was developed by István Lázár Jr., PhD, and István Lázár Sr., PhD, CSc, at the University of Debrecen.

### 3.4. Cleavage Site Identification by MALDI-TOF MS

The identification of cleavage positions was carried out by determining the proteolytic fragments’ molecular weight experimentally, followed by comparison of the measured values and the computed molecular weights. The cleavage of casein was carried out by using the reaction conditions described above, but the reaction mixtures were incubated for 90 min rather than only for 60 min, which ensured cleavages even at the less-preferred sites as well.

An Autoflex Speed matrix-assisted laser desorption/ionization time-of-flight (MALDI-TOF) mass spectrometer (Bruker Daltonik, Bremen, Germany) was used to analyze the substrates and cleavage fragments. The linear mode was used with 19.5 kV and 18.3 kV as ion source 1 and source 2 voltages. Peptide protein1 and protein2 calibration mixtures (Bruker Daltonik, Bremen, Germany) were used for external calibration. The spectra were evaluated by the flexAnalysis 3.4 software (Bruker). C4 Zip Tip pipette tips were applied to concentrate and desalt the samples before MS analysis. A 2,5-dihydroxybenzoic acid (DHB, 100 mg/mL) matrix solved in 50% aqueous acetonitrile with 0.1 V/V% trifluoro acetic acid (TFA) content was used. Then, a 1 µL sample and 0.5 µL matrix solution were mixed on the plate and allowed to air-dry.

## 4. Conclusions

Bovine casein is one of the most widely applied universal protease substrates, with both labelled and non-labelled forms available. It has already been utilized successfully to investigate activity of SARS-CoV-2 Mpro, although it has not been characterized as a substrate of Mpro, and the cleavage sites of Mpro remained to be determined. In this work, we studied α-, β-, and κ-isoforms of bovine casein and found that only β-casein is cleaved by SARS-CoV-2 Mpro in vitro. In this work, the cleavage sites were analyzed by using both in silico and in vitro approaches, revealing previously unknown cleavage site sequences. The experimental analysis of cleavage fragments did not reveal the release of free tyrosine [20,21,22]; rather, we observed cleavages next to glutamine residues, in agreement with the well-established specificity of Mpro, i.e., strong preference for a glutamine residue in the P1 position [25]. The NetCorona [24] and 3CLP [25] in silico approaches we used to identify the Mpro cleavage sites were found to have sufficient prediction potential; nevertheless, the cleavage site sequences we identified are expected to potentially aid the development of prediction algorithms and/or the improvement of already existing ones. In addition, the experimentally identified bovine β-casein cleavage sites further extend the knowledge about the specificity of SARS-CoV-2 Mpro.

Casein is a general whole protein substrate that can be cleaved by a wide range of proteases, including SARS-CoV-2 Mpro. The cleavage product formation can be followed by colorimetry and gel-electrophoresis, methods that are easy-to-perform and cheap, and there is no need for expensive instrumentation. In addition, our results obtained from the cleavage of a total casein isolate revealed that the presence of other isoforms (such as α- and κ) does not interfere with the processing of β-casein by Mpro. Accordingly, the use of more expensive purified β-casein fractions is not necessarily required, further increasing the cost-efficiency of a casein-based assay. Nevertheless, a possible limitation of casein-based assays might be a relatively lower sensitivity as compared to modified caseins (such as azocasein or FITC-labeled casein), fluorescent protein-tagged proteins [11,12], synthetic oligopeptides [38] or fluorophore-labelled peptide substrates [40]. However, the coloration or autofluorescence of complex biological and crude samples such as plant extracts [22] potentially causes interference with detection in spectrophotometric or fluorometric assays; in these cases, the use of SDS-PAGE might be advantageous to follow product formation. Accordingly, and due to its easy-to-use and cheap nature, casein is still a valuable substrate that can be efficiently utilized by simple experimental setups, e.g., for in vitro screening of candidate inhibitors of SARS-CoV-2 Mpro, in a cost- and time-efficient manner.

Besides the significance of casein as a general protease substrate, proteolytic digestion of casein in dairy milk and milk products is also of a special importance. For example, bovine breeds exhibit differences in the production of casein isoforms, such as that of A1 and A2 β-casein [41]. The digestion of β-casein results in the formation of degradation products referred to as β-casomorphins (BCMs); one of the most important is the hydrophobic heptapeptide BCM-7. A1 and A2 caseins exhibit a characteristic difference between their sequences, i.e., whether the C-terminal Ile residue of BCM-7 is followed by Pro or His in A2 and A1 casein, respectively (Tyr-Pro-Phe-Pro-Gly-Pro-Ile-His/Pro). Cleavage does not occur between P1-Ile and P1’-Pro residues in A2 β-casein, while A1 β-casein is susceptible for cleavage if a His residue replaces P1’-Pro in this position [42]. Accordingly, BCM-7 is produced only during digestion of A1 but not A2 β-casein. Some people have increased sensitivity against this peptide which is thought to correlate with inflammatory response in the digestive system and abdominal discomfort [43,44]. SARS-CoV-2 Mpro exhibits a strong preference for the cleavage site sequences that contain Gln in the P1 position, but it can also cleave non-canonically next to His residues [29]. The BCM-7 peptide is free from any Gln or His residue. During digestion of dairy milk, A1 β-casein can be cleaved between P1-Ile (the C-terminal residue of BCM-7) and P1’-His residues. However, this His residue is not expected to occupy the P1 position, and Mpro unlikely cleaves at the C terminus of BCM-7 (between P1-His and P1’-Asn residues), but such cleavage site sequence has not been identified yet [29]. Consequently, cleavage of β-casein isoforms by SARS-CoV-2 Mpro in vitro may not be applicable to differentiate A1 and A2 β-casein in the context of BCM-7 peptide.

High-resolution profiling of SARS-CoV and SARS-CoV-2 Mpro confirmed the maintenance of a nearly identical substrate specificity, despite minor sequence variations, underscoring its conserved function across coronaviruses and informing broad-spectrum inhibitor design [45]. The data obtained from the analysis of non-specific substrates might provide information about the interactions of SARS-CoV-2 Mpro with host proteins. For instance, research has identified several human proteins as substrates of Mpro, suggesting that the protease may modulate host cellular functions to facilitate viral replication. The conservation of its substrate recognition motifs suggests that inhibitors developed against SARS-CoV-2 Mpro may also be effective against other coronaviruses, offering a strategic advantage in pandemic preparedness. Additionally, the development of ubiquitin variants that inhibit the papain-like protease of SARS-CoV-2 has shown promise in reducing viral replication [46], highlighting potential therapeutic avenues. These insights into the substrate specificity and inhibition of SARS-CoV-2 proteases contribute to a more comprehensive understanding of viral–host interactions and may inform future antiviral strategies.

## Figures and Tables

**Table 1:** Comparison of the theoretical and measured molecular weights of β-casein and its cleavage fragments. The theoretical molecular weights were calculated based on protein sequence, while the measured values were obtained from MALDI-TOF MS analysis. * The molecular weight of non-cleaved β-casein is shown as the average of sequence variants’ molecular weight (Table S2). Cleavage site sequences are indicated. ** Cleavage fragments identified with the highest intensities.

Cleavage Position(s)	Cleavage Site Sequence	Theoretical	Measured
β-casein *		24,001	24,045
71	FAQTQ*SLVYP	17,057	17,079
		6944	6965
87	NSLPQ*NIPPL	15,349	15,308
156	LPLLQ*SWMHQ	16,349	16,373
		7652	7658 **
182	LSLSQ*SKVLP	4700	4687
71 + 190	LPVPQ*KAVPY	13,225	13,250 **
94 + 190	PPLTQ*TPVVV	10,753	10,770 **

**Table 2:** The enzyme, substrates and inhibitors used in this study. The SARS-CoV-2 Mpro was expressed and purified as described previously. Casein products were ordered from Sigma-Aldrich (St. Louis, MO, USA). * Casein sodium salt contains α-S1-, α-S2-, β- and κ-isoforms of bovine casein. ** SARS-CoV-2 Mpro was expressed and purified in this work based on previously published protocols [38].

Protein	Manufacturer	Source	UniProt ID
casein sodium salt (α-S1, α-S2, β, κ) *	Sigma-Aldrich, C8654	bovine milk	-
α-casein (α-S1, α-S2)	Sigma-Aldrich, C6780	bovine milk	P02662 (α-S1) P02663 (α-S2)
β-casein	Sigma-Aldrich, C6905	bovine milk	P02666
κ-casein	Sigma-Aldrich, C0406	bovine milk	P02668
SARS-CoV-2 Mpro **	in-house stock	recombinant	P0DTC1

## Data Availability

The datasets generated during and/or analyzed during the current study are available from the corresponding authors on reasonable request.

## Supplementary Materials

The following supporting information can be downloaded at: https://www.mdpi.com/article/10.3390/ijms26125829/s1.
