# Supplementary material for: Identification of SARS-CoV-2 Main Protease Cleavage Sites in Bovine β-Casein
Source: Int J Mol Sci. 2025 Jun 18;26(12):5829. doi: 10.3390/ijms26125829 (PMC12192567; doi:10.3390/ijms26125829)

Supplementary figure S1. Structure of bovine β-casein and the predicted cleavage sites of SARS-CoV-2 Mpro. The structural coordinates of β-casein modeled by AlphaFold were accessed through UniProt database (https://alphafold.ebi.ac.uk/entry/P02666) (date of last accession: 2023.08.03). Stick representation was used to highlight the phosphorylated serines and the P1-Gln residues of the cleavage sites. Color code: grey: β-casein; light red: P5-P5′ residues of predicted cleavage site sequences; red: P1-Gln residues of cleavage sites; blue: phosphorylation sites based on UniProt database.

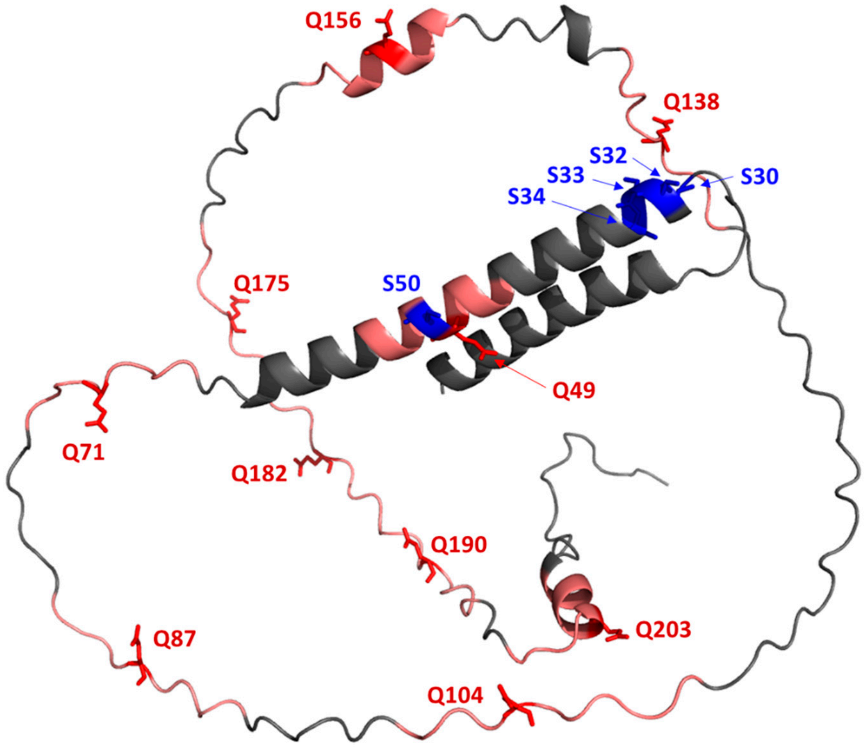

Supplement: Supplementary file 1 [file ijms-26-05829-s001.zip › Supplementary Figure S1.pdf]
