# Supplementary material for: Identification of SARS-CoV-2 Main Protease Cleavage Sites in Bovine β-Casein
Source: Int J Mol Sci. 2025 Jun 18;26(12):5829. doi: 10.3390/ijms26125829 (PMC12192567; doi:10.3390/ijms26125829)
Supplement: Supplementary file 1 [file ijms-26-05829-s001.zip › Supplementary Table S1.pdf]

Supplementary Table S1. Prediction of SARS-CoV-2 Mpro cleavage sites in casein isoforms. The sequences of bovine (*Bos taurus*) casein isoforms were downloaded from UniProt database, the identifiers are shown for each. The *in silico* prediction of SARS-CoV-2 Mpro cleavage sites was performed by using NetCorona 1.0 and 3CLP web servers (date of last accession: 2023.07.31). The default threshold of prediction is 0.5, the sites having <0.5 score are not considered to be cleavage site, the ≥0.5 probability implies possible cleavage. The higher prediction score the higher cleavage probability. The values between 0.4-0.5 and >0.5 are bold+italics and bold+underlined, respectively. Res: P1 residue.

			NetCorona 1.0		3CLP	
	**Res.**	**Sequence**	**Score**	**Cleavage**	**Score**	**Cleavage**
**P02666|CASB_BOVIN Beta-casein**	49	EKFQ*SE	***0.427***	***no***	0.39	no
	53	SEEQ*QQ	0.075	no	0.02	no
	54	EEQQ*QT	0.074	no	0.00	no
	55	EQQQ*TE	0.072	no	0.00	no
	61	DELQ*DK	0.129	no	0.11	no
	69	PFAQ*TQ	0.070	no	0.09	no
	71	AQTQ*SL	0.098	no	0.38	no
	87	SLPQ*NI	0.093	no	0.16	no
	94	PLTQ*TP	0.062	no	0.10	no
	104	PFLQ*PE	0.091	no	0.34	no
	138	TESQ*SL	0.221	no	0.35	no
	156	PLLQ*SW	***0.482***	***no***	**0.81**	**yes**
	161	WMHQ*PH	0.069	no	0.01	no
	164	QPHQ*PL	0.064	no	0.01	no
	175	FPPQ*SV	0.145	no	0.35	no
	182	SLSQ*SK	0.208	no	**0.52**	**yes**
	190	PVPQ*KA	0.068	no	0.00	no
	197	PYPQ*RD	0.073	no	0.17	no
	203	MPIQ*AF	0.121	no	***0.41***	***no***
	209	LLYQ*EP	0.058	no	0.01	no

			NetCorona 1.0		3CLP	
	**Res.**	**Sequence**	**Score**	**Cleavage**	**Score**	**Cleavage**
**P02668|CASK_BOVIN Kappa-casein**	22	LGAQ*EQ	0.059	no	0.01	no
	24	AQEQ*NQ	0.224	no	0.04	no
	26	EQNQ*EQ	0.064	no	0.00	no
	28	NQEQ*PI	0.064	no	0.00	no
	50	IPIQ*YV	0.080	no	0.10	no
	65	NYYQ*QK	0.078	no	0.07	no
	66	YYQQ*KP	0.060	no	0.00	no
	75	INNQ*FL	0.064	no	0.03	no
	93	SPAQ*IL	0.090	no	0.02	no
	96	QILQ*WQ	0.079	no	0.13	no
	98	LQWQ*VL	0.073	no	0.04	no
	110	KSCQ*AQ	0.102	no	0.07	no
	112	CQAQ*PT	0.081	no	0.00	no
	135	KKNQ*DK	0.080	no	0.01	no
	184	NTVQ*VT	0.101	no	0.13	no

			NetCorona 1.0		3CLP	
	Res.	Sequence	Score	Cleavage	Score	Cleavage
P02662|CASA1_BOVIN Alpha-S1-casein	24	IKHQ*GL	0.091	no	0.13	no
	28	GLPQ*EV	0.066	no	0.02	no
	67	TEDQ*AM	0.289	no	0.12	no
	74	DIKQ*ME	0.068	no	0.00	no
	93	SVEQ*KH	0.075	no	0.01	no
	97	KHIQ*KE	0.066	no	0.05	no
	112	YLEQ*LL	0.063	no	0.00	no
	123	KVPQ*LE	0.065	no	0.00	no
	145	IHAQ*QK	0.100	no	0.07	no
	146	HAQQ*KE	0.063	no	0.01	no
	155	GVNQ*EL	0.064	no	0.01	no
	167	LFRQ*FY	0.063	no	0.01	no
	170	QFYQ*LD	0.065	no	0.03	no
	187	LGTQ*YT	0.066	no	0.00	no

			NetCorona 1.0		3CLP	
	Res.	Sequence	Score	Cleavage	Score	Cleavage
P02663|CASA2_BOVIN Alpha-S2-casein	32	IISQ*ET	0.068	no	0.00	no
	37	TYKQ*EK	0.089	no	0.05	no
	94	KHYQ*KA	0.072	no	0.01	no
	102	EINQ*FY	0.064	no	0.00	no
	105	QFYQ*KF	0.062	no	0.00	no
	109	KFPQ*YL	0.062	no	0.01	no
	112	QYLQ*YL	0.080	no	0.10	no
	116	YLYQ*GP	0.064	no	0.18	no
	126	PWDQ*VK	0.122	no	0.06	no
	142	NREQ*LS	0.082	no	0.00	no
	184	KISQ*RY	0.064	no	0.00	no
	187	QRYQ*KF	0.089	no	0.00	no
	193	ALPQ*YL	0.069	no	0.02	no
	200	TVYQ*HQ	0.119	no	0.13	no
	202	YQHQ*KA	0.073	no	0.00	no
	210	PWIQ*PK	0.223	no	0.20	no
