# Supplementary material for: Identification of SARS-CoV-2 Main Protease Cleavage Sites in Bovine β-Casein
Source: Int J Mol Sci. 2025 Jun 18;26(12):5829. doi: 10.3390/ijms26125829 (PMC12192567; doi:10.3390/ijms26125829)
Supplement: Supplementary file 1 [file ijms-26-05829-s001.zip › Supplementary Table S2.pdf]

Supplementary Table S2. Sequence variants of bovine β-casein. The missense sequence variations of bovine β-casein were downloaded from UniProt database. A2 indicates the canonical sequence of β-casein (UniProt ID: P02666). Molecular weights (Mw) were calculated for the proteins lacking the N-terminal signal sequence. *Phosphorylation site (phosphoserine). **These residues constitute a part of a predicted cleavage site sequence (P4-P4′ residues), therefore, their mutation may potentially influence cleavage efficiency at the given sites. The calculated molecular weights of β-casein variants does not include those of the phosphate groups in the case of phosphorylated S30, S32, S33, S34 and S50 residues. "No of pSer" indicated number of phosphorylated serine residues. Mw+pSer indicated the molecular weight of phosphorylated isoforms.

		**β-casein residues**															
		**S33***	**R40**	**E51****	**E52****	**P82**	**L103****	**H121**	**E132**	**S137****	**L152**	**P153****	**P167**	**Q190**	**Mw (Da)**	**No. of pSer**	**Mw+pSer**
**Variant**	**A2**														23583.29	5	23983.19
	**A1**					H			Q		P	L		E	23623.31	5	24023.21
	**A3**							Q							23574.27	5	23974.17
	**B**					H				R					23692.42	5	24092.32
	**C**				K	H									23622.37	5	24022.27
	**D**	K													23624.38	4	23944.30
	**E**			K											23582.34	5	23982.24
	**F**					H							L		23639.35	5	24039.25
	**G**					H			Q			L		E	23639.35	5	24039.25
	**H**		C				I				P	L			23530.24	5	23930.14
**Average Mw (Da)**															23611.13 ± 44.92		24003.03 ± 49.21
